# Accidental Intrathecal Tranexamic Acid Injection During Caesarean Section: A Case Report

**DOI:** 10.1155/2024/4731010

**Published:** 2024-10-15

**Authors:** Raymond Oyugi Samuel, Victoria Adonicam, Andrew Hans Mgaya

**Affiliations:** ^1^Department of Anaesthesiology, Muhimbili National Hospital, Dar es Salaam, Tanzania; ^2^Department of Anaesthesiology, Muhimbili University of Health and Allied Sciences, Dar es Salaam, Tanzania; ^3^Department of Obstetrics and Gynaecology, Muhimbili National Hospital, Dar es Salaam, Tanzania

**Keywords:** bupivacaine, intrathecal injection, look-alike, medication error, tranexamic acid

## Abstract

**Background:** Tranexamic acid (TXA) is increasingly used in the management of haemorrhage during and after delivery and haemorrhage caused by other medical conditions due to its efficacy and safety. However, increasing report of fatal complications from inadvertent intrathecal TXA injection remains a cause of concern. The aim of this case report is to demonstrate clinical presentation and predictors of accidental intrathecal injection of TXA within the structure and processes of care in a health facility.

**Case Description:** A 37-year-old woman, multiparous woman presented with a diagnosis of obstructed labour and, therefore, was scheduled for emergency caesarean section. She was assigned the American Society of Anesthesiology II physical status. Spinal anaesthesia was performed at a sitting position through L4-L5 interspace using a 25-G spinal needle gauge. The anaesthetist injected 3 mL of an aesthetic agent that was prepared earlier as hyperbaric bupivacaine 0.5%. About 2 min after receiving the injection, the patient reported gluteal discomfort and itching and severe back pain. She subsequently developed progressive altered mentation followed by generalized tonic–clonic seizures. General anaesthesia was conducted with propofol (100 mg), pethidine (50 mg) and suxamethonium (100 mg). Episodes of tonic–clonic seizures continued despite treatment with multiple doses of diazepam (10 mg), propofol (100 mg) and phenytoin infusion (1 gm). Postoperatively, the patient was transferred to the intensive care unit with persistent tachycardia (125–138 beats per minute), hypertension (157/105–175/118 mmHg) and oxygen saturation of 90%–95%. She died due to cardiac arrest after 21 h of stay.

**Conclusion:** Medication error such as accidental intrathecal injection of TXA continues to jeopardise the safety of surgery under spinal anaesthesia.

**Summary**

• TXA is lifesaving yet imposes a risk of fatal medication error, when accidentally administered intrathecal instead of its look-alike, bupivacaine, during spinal anaesthesia.

## 1. Introduction

Tranexamic acid (TXA) is an antifibrinolytic drug discovered in 1960s and since then widely used to prevent bleeding by inhibiting activation of plasminogen to plasmin, thus stabilizing a haemostatic blood clot. Numerous studies reported TXA as a lifesaving medicine, which reduces blood loss and unnecessary morbidity and mortality [1, 2] if administered in correct dosage, route and time [3]. For these reasons, TXA is commonly found in cardiac, obstetrics, orthopaedics and trauma operating rooms [4]. In obstetric practice, the World Health Organization (WHO) recommends intravenous TXA within 3 h of birth as part of the standard treatment packages for postpartum haemorrhage (PPH) following vaginal births or caesarean section (CS), with a second dosage of 1 g IV if bleeding continues after 30 min [5]. Although extensively used for PPH prophylaxis during cesarean operation, there is still lack of evidence that TXA reduces mortality or need for blood transfusion during or after delivery [6]. Nevertheless, it is important to tailor patients' need. Some situations involving high-risk deliveries may justify prophylactic administration of TXA [7]. In addition to TXA valued use in the management of PPH, recent published evidence has shown that its use can extend outside labour ward and obstetric theatres. When used in bleeding patients' postemergency trauma, TXA was shown to reduce in-hospital mortality associated with haemorrhagic shock [8]. TXA's role in reducing haemorrhage during various surgical procedures among patients with bleeding disorders and those on antithrombotic agents has been established, making it a valuable drug in minimising the need for blood transfusion perioperatively [2].

However, medication errors associated with the use of TXA have been reported [9, 10], partly due to lack of adherence to a standardized system in preparing and administering medicines during anaesthesia. The standardized system includes, but not limited to, mandatory labelling of medicine and standardizing their location on the anaesthesia trolley and double-checking each medicine before use.

Accidental intrathecal injection of TXA instead of its “look-alike”—hyperbaric bupivacaine ampoule—is a tragic medication error [11], with subsequent fatal complications [5] during spinal anaesthesia. Reporting cases of adverse clinical outcomes due to medication errors remains essential in disseminating information that raises awareness of gaps and subsequent complications during clinical practice. We report a case of accidental intrathecal injection of TXA, instead of bupivacaine 0.5%, during spinal anaesthesia for emergency CS due to obstructed labour. The aim of this case report is to demonstrate clinical presentation and predictors of accidental intrathecal injection of TXA within the structure and processes of care in a health facility. This case report shall create an opportunity to improve surgical safety and link quality improvement (QI) interventions to relevant safety threats in anaesthesia and surgery in routine clinical practice.

## 2. Case Description

A 37-year-old woman, gravida five, para three with three living children was scheduled for emergency CS due to obstructed labour at full term. She was assigned the American Society of Anesthesiology (ASA) II physical status. Preanaesthetic survey was completed, and laboratory investigations performed were within normal limits. The informed written consent for CS was obtained. She arrived in the operating room awake and oriented. Her vital signs and foetal heartbeats were recorded to be normal.

Patient was placed at a sitting position with her feet resting on a stool. Her back was cleaned with povidone–iodine and alcohol after the L4-L5 interspace was located. A 25-G Quincke spinal needle was used to perform spinal anaesthesia under aseptic technique. CSF backflow was observed by the anaesthetist before injecting 3 mL of an anaesthetic agent that was prepared earlier as hyperbaric bupivacaine 0.5%. About 2 min later, the patient reported gluteal discomfort and itching, followed by severe back pain. Intravenous hydrocortisone (100 mg) was given without improvement. Another 2 min lapsed before the patient complained of chest symptoms and subsequently became agitation. Thereafter, she developed progressive altered mentation and subsequent generalized tonic–clonic seizures. There were no reported signs of sensory or motor block by then. Immediately, a decision to convert to general anaesthesia (GA) was made and rapid sequence induction was performed with propofol (100 mg), pethidine (50 mg) and suxamethonium (100 mg). Episodes of tonic–clonic seizures were unsuccessfully treated with two doses of diazepam (10 mg) and propofol (100). CS was performed after seizures were transiently arrested with phenytoin (1000 mg) infusion. An empty ampoule of TXA was later found in the same tray with unused hyperbaric bupivacaine 0.5% hence ascertaining intrathecal TXA administration.

The patient was transferred to the intensive care unit (ICU) for postoperative care on mechanical ventilation through an endotracheal tube. In the ICU, the patient continued developing repeated episodes of seizures that were unresponsive to phenytoin (1000 mg) and midazolam (5 mg) infusions. The patient monitors recorded tachycardia (125–138 beats per minute), hypertension (157/105–175/118 mmHg) and oxygen saturation of 90%–95% on mechanical ventilation. The body temperatures were within normal range (36.3^o^C–37.1°C). Serum glucose and electrolyte levels were also within normal limits. The patient succumbed following unsuccessful resuscitation due to cardiac arrest and clinically evident acute kidney injury after 21 h of stay in the ICU.

## 3. Discussion

TXA is a lifesaving medicine that continues to impose potential risk of medication error with fatal consequences, hence the need for addressing this problem in all operating rooms. Similar to our case report, other reported cases of accidental intrathecal injection of TXA have increasingly conformed to a typical clinical presentation comprising of gluteal itch and pain, back pain, altered mental status, refractory seizures, hypertension, ventricular fibrillation and death [4, 11, 12]. Thus, awareness of typical clinical presentation of these adverse reactions enables early diagnosis and intervention without undermining the importance of implementing measures for preventing this fatal medication error. TXA, the most commonly used antifibrinolytic, is known to cause seizures. Theoretically, TXA can cause neurotoxicity, as a result of heightened excitability of neurons, when bound to alpha aminobutyric acid type A and glycine receptors in the spinal dorsal horn nuclei. Intractable convulsions may be attributed by suppression of inhibitory neurotransmission as well as cerebral vasoconstriction, ischaemia and raised intracranial pressure [13–17]. In addition, experimental studies in animals have shown that application of TXA in the brain cortex causes generalised seizures [14]. Hypertensive reaction and subsequent ventricular arrhythmia can result from massive sympathetic discharge following the high dosage of TXA [18], which may be what happened in our case.

Mohsen and Sabzi [19, 20] reported two obstetric cases of similar medication error during CS. Similar to our case, these cases also presented with generalized seizures and refractory ventricular fibrillation that were followed by episodes of cardiac arrest and death. Nonetheless, effort for medical and mechanical defibrillation was also futile. Al-Taei and colleagues [10] reported another medication error with typical clinical features comprising myoclonic seizures and hypertension. Despite the effort of endotracheal intubation and sedation with intravenous levetiracetam (1000 mg) and midazolam infusion (5 mg), the patient died in the ICU due to respiratory failure. An incident of accidental intrathecal injection of 200 mg of TXA that involved a patient with preeclampsia [9] was also reported trailing a similar clinical pathway as other case reports before suffering respiratory failure and cardiac arrest.

Even though a pregnancy state tends to increase sensitivity to intravenous induction and sedative agents [21], our case and other reviewed cases of accidental intrathecal TXA developed generalized seizures that were unresponsive to anticonvulsants and sedatives such thiopental (300 mg), phenytoin (1000 mg) and midazolam (5 mg) infusions. The use of inhalation anaesthetic agents during CS might have reduced the potency of anticonvulsant medicines [22]. Nonetheless, there was a risk of aspiration during initial episodes of seizure, before endotracheal intubation, and ultimately respiratory failure and death. Physiological decrease in CSF volume in pregnancy might have contributed to a relatively high concentration of TXA in the CSF [23], hence increasing the risk of the treatment failure and mortality.

Whilst Harby and Kohaf [11] reported successful treatment of accidental intrathecal TXA by employing CSF lavage, Kaabachi and colleagues [24] treated a similar case without performing CSF lavage.

For our case, early CSF lavage could have minimized adverse complications by dilution and elimination of TXA. However, the rarity of this particular medication error could have lowered the index of suspicion of accidental intrathecal injection of TXA, following a routine spinal anaesthesia. Thus, it was unlikely to perform CSF lavage promptly. Considering the high risk of mortality associated with intrathecal TXA, the benefits of CSF lavage outweigh the risks associated with the removal and replacement of 120–150 mL of CSF [12, 24]. Therefore, there is a need to continue raising awareness of healthcare providers of typical clinical presentation and circumstances leading to accidental intrathecal injection of TXA.

High risk of mortality associated with intrathecal TXA is reported by numerous authors including Patel and colleagues [4] who identified 21 cases of intrathecal injection between 1960 and 2018, of which almost all developed life-threatening state and 50% died. Similar to our case, accidental swapping of TXA for bupivacaine was associated with “look-alike” ampoule medication error [9, 11]. TXA is commonly administered via oral or intravenous route and in high doses. Most of the side effects reported in humans are tolerable and these include gastrointestinal upsets, headache and muscle aches [17]. Since the benefits of TXA in reducing mortality due to haemorrhage outweigh the risks associated with its use in the operating room, then strategies to prevent medication error should focus on minimizing or eliminating the risk of accidental swapping and misreading medicines during anaesthesia.

In our case, lack of adherence to guidance for handling anaesthetic agents contributed to the fatal medication error, as it happens in all parts of the world [4, 5, 9–12]. Despite a specialist-led anaesthesia team and the presence of guidelines for anaesthetic process, proper sorting and labelling of medicines and double-checking before administration of anaesthetic agents were likely omitted. Hence, swapping or misreading of TXA ampoule as its look-alike hyperbaric bupivacaine 0.5% ampoules was almost inevitable. Therefore, continuous dissemination of information with regard to this medication error through publications, workshops and conferences remains essential in sensitizing care providers the necessity of proper sorting and labelling medicines and meticulous drug identification checks.

## 4. Conclusions and Recommendations

Medication error such as accidental intrathecal injection of TXA continues to jeopardize the safety of surgery under spinal anaesthesia. There is high likelihood of unintentional swapping of TXA for bupivacaine 0.5% when both medicines are placed on the same tray. The risk can extend to other medication errors, if double-checking medicines is omitted and in the case of dysfunctional teamwork when conducting anaesthesia. High index of suspicion and early appropriate treatment of adverse complications can reduce the risk of unnecessary mortalities.

The following recommendations were made by the WHO/FDA [5] and the Anaesthesia Patient Safety Foundation (APSF) [25] to healthcare professionals and other stakeholders when handling TXA:

a. Storage of TXA away from the anaesthetic drug trolley, preferably outside the room.
b. Store TXA injection vials separately from other drugs in a way that makes the labels visible to avoid reliance on identifying drugs by the vial cap colour.
c. Add an auxiliary warning label to note that the vial contains TXA.
d. Check the container label to ensure the correct product is selected and administered.
e. Restricting or eliminating TXA vials/ampules and only allowing manufacturer-prepared ready-to-administer TXA 1000 mg-per-100 mL bags or pharmacy-prepared IV bags as per APSF conference recommendation. Ready-to-administer TXA is considered most realistic and achievable means to prevent TXA–bupivacaine errors.
f. Different packaging and labelling of TXA and bupivacaine by manufacturers.
g. Utilize barcode scanning when stocking medication cabinets and preparing or administering the product.

The authors further recommend the following:

a. Implementing strategies to improve adherence to standard guideline for providing spinal anaesthesia including proper labelling and sorting of all drugs on the anaesthesia trolley, double-checking practices and preparedness for employing GA in anticipation of failure or adverse complications of spinal anaesthesia.
b. Employing institutional written policies that integrate quality-improvement framework within audit and feedback of every medication error, thus building a culture of drug safety in all disciplines by changing structure and process of care towards minimizing the possibility of occurrence of medication errors.
c. Imparting knowledge and skills of ABC protocol for operating room staff as a primary intervention in improving management of accidental intrathecal injection of TXA. Moreover, staff should also be trained to perform continuous CSF lavage and how to select appropriate sedative agents with neural protection for ICU patients.
d. Extensively disseminating information that raises awareness of typical clinical presentations and complications of intrathecal TXA and establishing local hospital protocols to ensure that TXA is stored separately from another anaesthetic agent, preferably outside the operating room and with an auxiliary warning label.
e. Evaluating workload to ensure workload pressures will not result in unsafe workarounds and practices.

## Data Availability

Data will be available from the corresponding author upon request.

## Nomenclature

ASA: American Society of Anesthesiologists
CS: Caesarean section
CSF: Cerebrospinal fluid
FDA: Food and Drug Administration
GA: General anaesthesia
ICU: Intensive care unit
iv: Intravenous
TXA: Tranexamic acid
WHO: World Health Organization

## References

[1] Shakur H., Roberts I., Fawole B (2017). Effect of Early Tranexamic Acid Administration on Mortality, Hysterectomy, and Other Morbidities in Women With Post-Partum Haemorrhage (WOMAN): An International, Randomised, Double-Blind, Placebo-Controlled Trial. *The Lancet*.

[2] Ockerman A., Vanassche T., Garip M. (2021). Tranexenamic Acid for the Prevention and Treatment of Bleeding in Surgery, Trauma and Bleeding Disorders: A Narrative Review. *Thrombosis Journal*.

[3] Colomina M. J., Contreras L., Guilabert P., Koo M., Mndez E., Sabate A. (2022). Clinical Use of Tranexamic Acid: Evidences and Controversies. *Brazilian Journal of Anesthesiology (English Edition)*.

[4] Patel S., Robertson B., McConachie I. (2019). Catastrophic Drug Errors Involving Tranexamic Acid Administered During Spinal Anaesthesia. *Anaesthesia*.

[5] Moran N. F., Bishop D. G., Fawcus S (2023). Tranexamic Acid at Cesarean Delivery: Drug-Error Deaths. *International Journal of Gynecology & Obstetrics*.

[6] Pacheco L. D., Clifton R., Saade G (2023). Tranexamic Acid to Prevent Obstetrical Hemorrhage After Cesarean Delivery. *New England Journal of Medicine*.

[7] Bouvet L., Rackelboom T., Bouthors A. S (2023). Does Tranexamic Acid Have Its Place in the Prevention of Postpartum Hemorrhage. *Anaesthesia Critical Care & Pain Medicine*.

[8] Al-Jeabory M., Szarpak L., Attila K. (2021). Efficacy and Safety of Tranexamic Acid in Emergency Trauma: A Systematic Review and Meta-Analysis. *Journal of Clinical Medicine*.

[9] Shah P. J., Agrawal P., Nagaria A., Habeeba Kp U. (2021). Fortuitous Intrathecal Injection of Tranexamic Acid. *Indian Journal of Anaesthesia*.

[10] AL-Taei M. H., AlAzzawi M., Albustani S., Alsaoudi G., Costanzo E. (2021). Incorrect Route for Injection: Inadvertent Tranexamic Acid Intrathecal Injection. *Cureus*.

[11] Harby S. A., Kohaf N. A. (2023). Accidental Intrathecal Injection of Tranexamic Acid: A Case Report. *Journal of Medical Case Reports*.

[12] Karl Matthew C Sy S. (2021). Accidental Intrathecal Administration of Tranexamic Acid: A Case Report. *Journal of Clinical Anaesthesia Pain Management*.

[13] Goyal G., Vajpayee A., Kant R., Singh R. (2014). Refractory Status Epilepticus After Accidental Intrathecal Injection of Tranexamic Acid. *Journal of Acute Medicine*.

[14] Lecker I., Wang D. S., Whissell P. D., Avramescu S., Mazer C. D., Orser B. A. (2016). Tranexamic Acid-Associated Seizures: Causes and Treatment. *Annals of Neurology*.

[15] Ohashi N., Ohashi M., Endo N., Kohno T. (2017). Administration of Tranexamic Acid to Patients Undergoing Surgery for Adolescent Idiopathic Scoliosis Evokes Pain and Increases the Infusion Rate of Remifentanil during the Surgery. *PLoS One*.

[16] Srivastava U., Joshi K., Gupta V. (2012). Accidental Injection of Tranexamic Acid Into Subarachnoid Space Leading to Fatal Outcome: Case Report and Review. *The Internet Journal of Anesthesiology*.

[17] Mahmoud K., Ammar A. (2012). Accidental Intrathecal Injection of Tranexamic Acid. *Case Reports in Anesthesiology*.

[18] Murdaca G., Greco M., Vassallo C., Gangemi S. (2020). Tranexamic Acid Adverse Reactions: A Brief Summary for Internists and Emergency Doctors. *Clinical and Molecular Allergy*.

[19] Mohseni K., Jafari A., Nobahar M. R., Arami A. (2009). Polymyoclonus Seizure Resulting From Accidental Injection of Tranexamic Acid in Spinal Anesthesia. *Anesthesia & Analgesia*.

[20] Sabzi F., Teimouri H., Zokai A. (2009). Myoclonus, Seizure, and Ventricular Fibrillation After Intrathecal Injection of Tranexamic Acid. *Journal Tehran University Hear Center*.

[21] Christensen J. H., Andreasen F., Jansen J. A. (1981). Pharmacokinetics of Thiopental in Caesarian Section. *Acta Anaesthesiologica Scandinavica*.

[22] Gorsky K., Cuninghame S., Chen J (2021). Use of Inhalational Anaesthetic Agents in Paediatric and Adult Patients for Status Asthmaticus, Status Epilepticus and Difficult Sedation Scenarios: A Protocol for a Systematic Review. *BMJ Open*.

[23] Igarashi T., Hirabayashi Y., Shimizu R., Saitoh K., Fukuda H., Suzuki H. (2000). The Fiberscopic Findings of the Epidural Space in Pregnant Women. *Anesthesiology*.

[24] Kaabachi O., Eddhif M., Rais K., Zaabar M. A. (2011). Inadvertent Intrathecal Injection of Tranexamic Acid. *Saudi Journal of Anaesthesia*.

[25] Lefebre P. A., Meyer P., Lindsey A., Jew R., Rebello E. (2024). *Unraveling a Recurrent Wrong Drug-Wrong Route Error-Tranexamic Acid in Place of Bupivacaine: A Multistakeholder Approach to Addressing This Important Patient Safety Issue*.

